# Mental Stress Detection Using Physiological Sensors and Artificial Intelligence: A Review

**DOI:** 10.3390/s26051616

**Published:** 2026-03-04

**Authors:** Rabah Al Abdi, Shouq AlKaabi, Shada Elsifi, Jawad Yousaf

**Affiliations:** 1 Electrical, Computer, and Biomedical Engineering Department, Abu Dhabi University, Abu Dhabi 1790, United Arab Emirates; 2 Biomedical Engineering Department, Jordan University of Science and Technology, Irbid 22110, Jordan

**Keywords:** mental stress detection, wearable devices, real-time monitoring, physiological sensors, galvanic skin response (GSR), electrocardiogram (ECG), heart rate variability (HRV), pupil diameter (PD), photoplethysmography (PPG), electroencephalogram (EEG), artificial intelligence algorithms, non-invasive techniques, State-Trait Anxiety Inventory (STAI), Perceived Stress Scale (PSS-10), mental health monitoring

## Abstract

Stress can cause many disorders, including mental and physical ones, if it persists. To take timely and effective early intervention measures, mental stress levels must be carefully monitored. This study investigates the rapidly growing topic of mental stress detection, focusing on the primary goals and mechanisms of existing detection frameworks. The main objectives and mechanisms will be highlighted. This study examines physiological sensors, stressors, algorithms, monitoring methods, and validation tools used to assess and classify mental stress. The study targets physiological sensors. Wearable sensors are becoming more popular because they can continuously monitor physiological responses in human-like environments. This allows them to reveal relevant stress patterns across various work environments. Numerous physiological sensors are used regularly. Galvanic skin response (GSR), electrocardiogram (ECG), photoplethysmography (PPG), electroencephalography (EEG), and pupil diameter camera systems are examples of these sensors. The combination of these sensors provides a wealth of cognitive and autonomic response data for stress detection. This review examines AI-based methods for interpreting complex physiological data. Machine learning and ensemble models are emphasized for improving stress classification accuracy and reducing incorrect classifications. In addition, this article discusses stressors used to induce reliable physiological responses. Validated self-report instruments are being reviewed as benchmarking tools for objective sensor-based measurements. STAI and PSS-10 are examples. These instruments demonstrate a strong correlation between stress and anxiety and physiological health outcomes. In conclusion, this review discusses future research avenues, focusing on advanced artificial intelligence-driven approaches and sophisticated sensors. These developments aim to better define stress levels and physiological factors that have not been thoroughly studied.

## 1. Introduction

According to several studies, mental stress is linked to many psychological and physical issues [1]. Early detection of stress can help mitigate it and prevent the consequences of diseases [2]. The demand for real-time monitoring systems has made wearable systems popular [3,4,5]. Physiological characteristics linked to autonomic nervous system stress responses include pupil diameter (PD), heart rate (HR), HR variability (HRV), and galvanic skin responses (GSR). These physiological markers are linked to stress [6]. Wearable technologies with sensors can monitor these parameters while the subject performs daily tasks [3]. Multimodal stress detection improves accuracy by integrating multiple sensors to detect physiological stress markers [2,5,6]. The most common combination includes the heart rate (HR), respiratory rate (RR), and arterial oxygen saturation (SpO_2_).

Machine learning algorithms can improve stress detection systems. K-Nearest Neighbours (KNNs), Support Vector Machines (SVMs), Random Forests (RFs), and Artificial Neural Networks (ANNs) are various types of neural networks utilized in [6,7]. KNN and RF are effective methods for stress classification. This can be accomplished by analyzing proximity patterns. Classification errors can occur when high-dimensional data and noisy inputs are present [8,9,10]. This can make it more difficult for them to achieve their objectives and to reduce their efficiency. These circumstances are possible. Therefore, it is possible that their work is less effective. Stress classification is accomplished using hyperplanes and optimal kernel functions by support vector machine models [7,9,11]. ANN models can self-train to identify stress patterns in complex multidimensional data [7,12]. These models are more adaptable than traditional neural networks. Ensembles of two or more models have a higher classification accuracy, along with better sensitivity and specificity [2,6] than individual models at the cost of higher computational resources. By building on the strengths of each model, stress detection algorithms have become more robust [2,6]. Ensemble models provide stress monitoring systems with high sensitivity and specificity [2,6].

Wearable mental stress systems become important to detect stress. User comfort is crucial when designing noninvasive wearable sensor systems. This is because the subject’s comfort allows continuous monitoring without being intrusive [4,13]. In this study, we present a review of the trends, strengths, and weaknesses of wearable stress detection devices. This study also assessed the current state of the field. Wearable device data analysis has changed significantly due to advancements in AI algorithms. Detailed physiological data can be interpreted using machine learning models to improve detection accuracy. This can be achieved using AI. Integrated multi-sensor approaches and advanced AI could improve real-time stress detection [2,5]. The aim of this study was to guide mental health monitoring research by demonstrating how these methods can improve stress detection accuracy and utility. This will fulfill our goal of guiding future research and development efforts.

This literature review aims to analyze the sensors, methodologies, and validation approaches used for mental stress detection, with a particular focus on wearable systems. By examining current trends, strengths, and limitations across sensing technologies and analytical techniques, this review provides a comprehensive overview of the state of wearable stress detection research. The findings are intended to support the development of more accurate, robust, and adaptive monitoring systems, while guiding future research toward integrated, multimodal approaches that leverage advanced AI techniques for real-time mental stress detection.

Questionnaires are also used for stress evaluation. The State-Trait Anxiety Inventory (STAI) by Charles et al. [14] and the Perceived Stress Scale (PSS-10) by Cohen et al. [15] are stress-specific questionnaires that reflect mental stress. Both questionnaires were developed by different research groups. Their service verifies the stress detection accuracy of wearable monitoring devices [2,6]. Questionnaires are reliable for wearable monitoring results, according to several studies [2,16,17]. Other studies have supported these findings.

Unlike earlier reviews that primarily focused on Wearable stress detection studies published prior to 2021 [18] or concentrated exclusively on EEG-based approaches [19], this review extends the analysis to include underexplored physiological markers such as pupil diameter (PD) and pulse wave amplitude (PWA). Furthermore, it incorporates recent advances in ensemble and deep learning models reported between 2023 and 2025, achieving classification accuracies exceeding 98%, while explicitly evaluating wearability constraints and continuous real-time monitoring requirements for practical stress detection systems [2,8,20].

The remainder of this paper is organized as follows. Section 2 explains the methodology employed for the selection and evaluation of the reviewed studies. In-depth information regarding the numerous approaches and procedures utilized for the stress analysis is provided in Section 3. Section 4 provides an analysis of the methodologies and approaches that have been studied to effectively characterize stress using wearable sensors. Section 5 provides a cross-comparison of all the methodologies and techniques that were examined, as well as a discussion of their advantages and disadvantages. In Section 6, we provide a summary of the findings obtained from the study.

## 2. Methodology of Literature Search for Review and Evaluation

**Figure 1:**
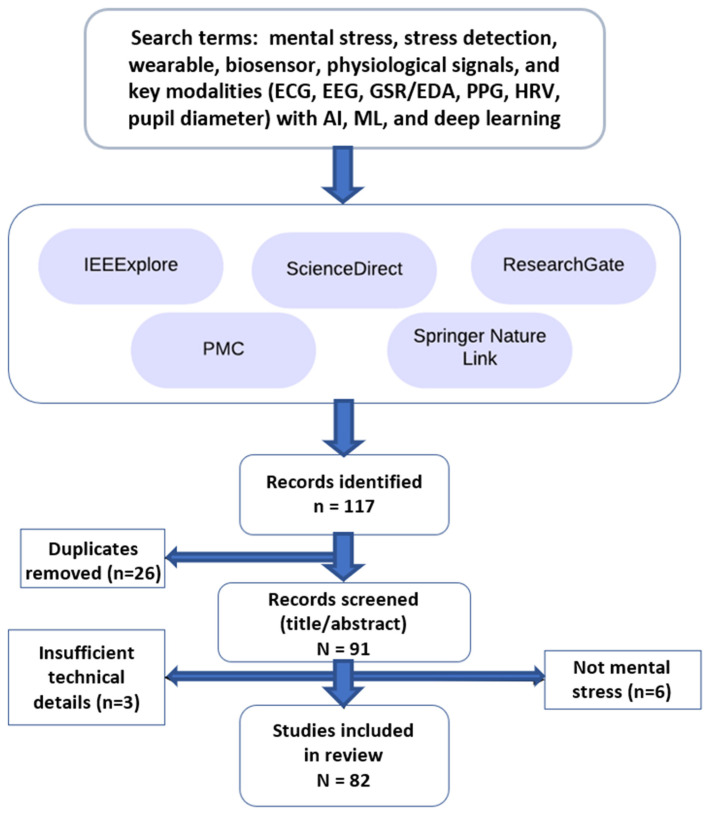
Reports the PRISMA flow with exclusion reasons at each step. Overall, 117 records were identified, 26 duplicates removed, and 91 unique records screened; 9 were excluded with documented reasons, resulting in 82 included studies.

We prioritized high-quality, resource-rich publications to develop an exhaustive and reliable literature review. This curated set of articles could support stress detection research. To ensure rigorous and relevant information, several inclusion criteria were applied. The overall study selection process is illustrated in Figure 1.

A structured search combined terms for mental stress/stress detection, wearable/biosensor/physiological signals, and key modalities (ECG, EEG, GSR, PPG, HRV, pupil diameter/pupillometry) with AI/ML/deep learning, applying minor syntax adjustments for each database (IEEE Xplore, ScienceDirect, Springer Nature Link, and PMC). ResearchGate was used only as a supplementary search with a simplified query and duplicates removed. These sources provide data-rich, peer-reviewed papers. To keep the review current and relevant, most of the research was published within the last five years (2019–2025). A small number of studies published before 2019 were included only if they were highly cited/seminal and directly relevant to wearable/physiological mental stress detection. This rule was applied consistently to avoid selective inclusion. Studies were included if they focused on mental/psychological stress, used physiological/biosignal measurements in a wearable or non-invasive (deployable) setup, and reported quantitative evaluation metrics (e.g., accuracy, sensitivity, specificity, F1, AUC) or sufficient methodological detail for comparison.

Studies were excluded if they focused only on physical stress/fatigue rather than mental/psychological stress, relied solely on questionnaires/self-reports without physiological sensing, lacked experimental validation or sufficient technical/methodological detail, or were non-English. Duplicate records were removed before screening, then titles/abstracts and full texts were assessed against these criteria.

## 3. Methods and Techniques for Stress Analysis

This section summarizes the primary methods for analyzing mental stress. This work discusses common stress-detection methods, physiological and behavioral stressors, validated questionnaires, artificial intelligence algorithms, and stress detection sensing modalities. This section discusses how methodological choices in laboratory and real-world settings affect stress assessment accuracy, dependability, and real-time applicability. This goal is achieved by reviewing these essential components in detail.

### 3.1. Techniques

**Figure 2:**
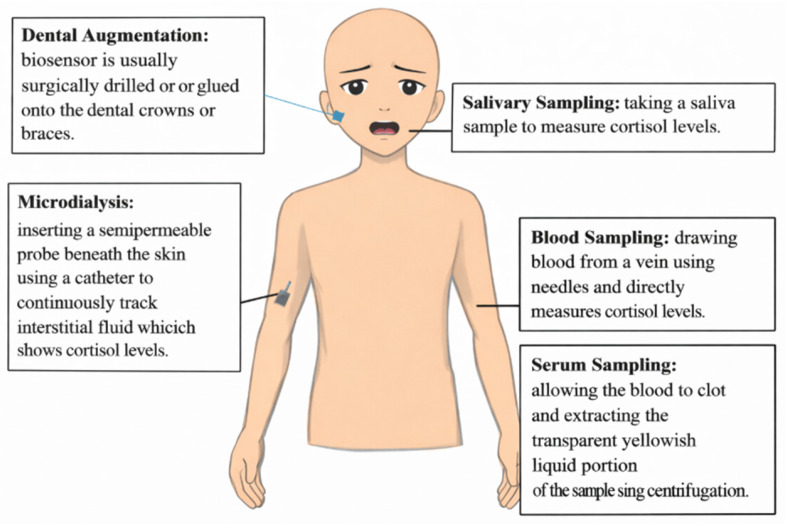
Invasive and minimally invasive cortisol sensing and sampling techniques.

**Figure 3:**
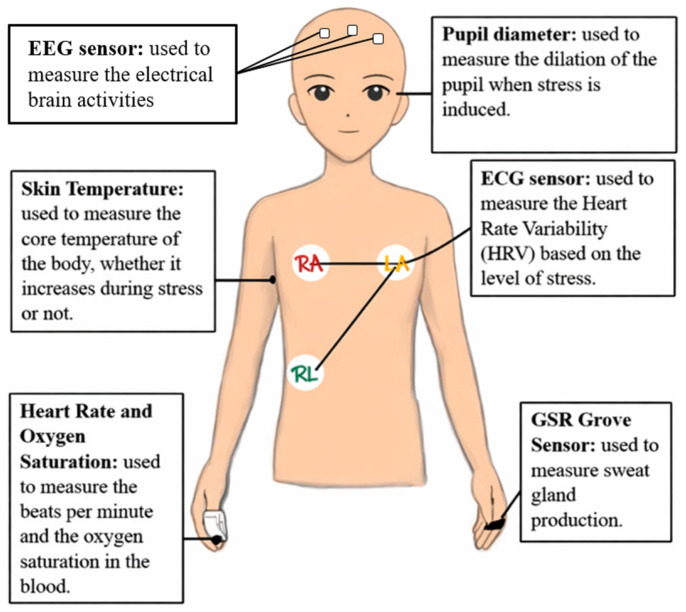
Non-invasive physiological sensor placements for stress monitoring.

Different physiological signals can be expressed, altered, and collected during stress. Figure 2 shows that metabolic processes related to mental stress are studied using invasive methods. Surgical insertion, venipuncture, and sampling through the mouth are invasive methods for obtaining biosensor-based measurements or collecting biological samples. Non-invasive procedures strive to avoid physical harm or obstruction, in contrast to invasive procedures. Consequently, they can reduce discomfort and may improve feasibility for repeated data collection, as shown in Figure 3. These methods can be modified depending on the physiological parameter being targeted, using electrocardiogram probes or oximeter modules, or by measuring eye pupil diameter. In summary, both methods provide enough information to distinguish stress classes. Researchers can study HRV and blood cortisol levels, which may yield valuable results [19,21].

From a deployment perspective, invasive techniques (e.g., blood/serum sampling, implanted probes) can provide high biochemical specificity and analytically precise measurements, which are valuable for short, controlled validation studies. However, these approaches typically impose higher participant burden, require clinical oversight and specialized infrastructure, and may reduce compliance in longitudinal or real-world monitoring. In contrast, non-invasive wearable sensing offers a more practical pathway for continuous stress monitoring because it is safer, more acceptable to participants, and easier to scale outside the laboratory. Recent advances in implantable sensor design aim to reduce invasiveness through miniaturized form factors, compliant sensor–tissue interfaces, and minimally invasive implantation approaches, yet these remain less feasible than wearables for broad, routine stress monitoring.

Fluid analysis-based cortisol monitoring applications were studied by Wangkhem et al. [22]. Since cortisol is the primary stress biomarker, this study was conducted. Blood sampling and biosensor insertion during dental augmentation were the most invasive procedures in their research. In dentistry, every tooth modification is called an “augmentation”. Dentures, crowns, implants, braces, and dentures are examples of dental restorations. Even so, the treatment is expected to hurt. In dental crowns or braces, the biosensor is drilled or glued to stay in place. Technical issues could cause discrepancies. Inaccurate biosensor placement and sensor quality can lower readings. However, the implant’s high sensitivity and continuous monitoring allow researchers to collect accurate data if performed correctly. They can then characterize mental stress caused by various factors [21].

In another invasive method, a small blood sample is drawn from patients via venipuncture during invasive research. This method helps assess mental stress by immediately measuring cortisol levels. Despite easy access to precise analytical information, this methodology may not cover all experimental procedures. While salivary biosensors can collect real-time data, blood samples take a long time to process in the lab. Thus, blood sample data collection is not a good way to measure stress continuously and in real time. Cross-contamination can also decrease the usability of stress measurement from blood sampling. A controlled environment is needed for experiments, but external factors can affect blood collection results. After needle insertion, physiological reactivity increases. This could cause stress levels to vary independently of stressors, producing unreliable data. Blood sampling can measure cortisol levels, but it is risky, time-consuming, and expensive [21].

The transparent, yellowish liquid sample that is collected from serum samples must be centrifuged in order to determine the cortisol level. As opposed to blood sampling, this analysis produces more reliable results. Every method has some downsides to it. There is a requirement for venipuncture, which is a lengthy medical procedure. A specialized laboratory and expensive technology are required in order to extract the components that are required. According to the results of the experiments, this method is not suitable for routine analysis [23]. One of the most effective methods for extracting cortisol is the use of serum sampling.

There is another method available for measuring cortisol, and that is microdialysis. Utilizing this method requires the placement of a semipermeable probe that is inserted through a catheter beneath the skin. Direct evaluation of cortisol activity and continuous monitoring of interstitial fluid are both possible with this method. Microdialysis is a less invasive method than serum sampling. Technical challenges that are common include complicated setups and the unpredictability of data. Pain can last for hours or even days [23]. The catheter and the probe are, as was to be expected, extremely uncomfortable [24]. Recent progress in implantable sensor design emphasizes minimally invasive implantation and compliant sensor–tissue interfaces to reduce tissue damage and foreign-body reactions, enabling more stable long-term in vivo monitoring. However, these approaches still require clinical implantation procedures and therefore remain less suitable for large-scale, routine stress monitoring compared with wearables.

Non-invasive methods can measure mental stress by measuring physiological signals with body sensors. Unlike invasive treatments, non-invasive methods protect the subject and ensure comfort while achieving satisfactory accuracy. They also enable real-time data analysis. Setting methods for feature extraction and technical settings is faster. External noise can confuse non-invasive methods. Sensor-based processes require excessive electronic instrumentation. This can be solved by signal filtering, which focuses on extracting important information from the recorded signal and reducing irrelevant activity [25].

Standard sensors for assessing mental stress include electrocardiogram (ECG), photoplethysmography (PPG), galvanic skin resistance (GSR), and electroencephalogram (EEG). Emerging skin-like, ultrathin dry electrodes are being developed to improve long-term signal quality and reduce motion-related distortions by maintaining stable skin contact and lowering noise during dynamic monitoring. Such materials can enhance wearable electrophysiological sensing (e.g., ECG/EEG) while improving comfort and adherence in real-world use [26]. An algorithmic computation analysis of each module allowed Zhou et al. to accurately distinguish stressed participants from those who were not [25].

According to Hanshans et al. [27], real-time stress oscillations and non-invasive wearable sensor feature extraction are related. Their correlations are related to the same phenomenon. This study evaluated the accuracy, consistency, ease of use, noise tolerance, and duration of EEG, GSR, peripheral skin temperature (PST), and heart rate variability (HRV). The participants were stressed by virtual reality. This was done by flipping the horror film 180 degrees. Participants are fully immersed in virtual reality, making this method effective. The data collection process is less likely to be affected by non-experimental factors. EEG, GSR, and PST showed physiological differences between stress and non-stress environments. The participant’s HRV changed slightly after watching a horror movie, which was contrary to expectations. Therefore, it was removed from the final parameters investigated. This study used non-invasive methods to distinguish stress and non-stress parameters, yielding reliable results. Each sensor displays mental stress-related physiological changes. Their analysis and experimentation have drawbacks. Insufficient sensor data extraction time and noise interference that disrupts readings are examples of drawbacks. Nevertheless, these sensors monitor and reduce major risks produced by invasive methods [27].

Direct contact in shared spaces increased COVID-19 transmission, harming at-risk individuals. Data retrieved outside of the controlled stressor may be inaccurate because people may not know the risk of infection. Despite considering every safety procedure, this is the case. Baran et al. used thermal imaging to monitor stress without contact or intrusion to reduce its risks [28]. Reduced risks were the reason. This technology allows secure, comfortable real-time data collection and measurement. This method is accessible and cheaper than EEG and other sensors. Thermal imaging may be limited by stress-related physiological changes. This is because thermal imaging uses temperature variations. The experiment must be done in a temperature-controlled environment to minimize the impact of such interference [28].

### 3.2. Stressors

To accurately characterize the physiological changes that are associated with mental stress, it is necessary to use an adequate set of stress inducers. When taking into account the characteristics of the experiment, it is essential to identify the method that will elicit the strongest and most reproducible stress response in order to achieve the desired results. The establishment of a correlation between the parameters that were recorded and the levels of stress is extremely important, particularly because stress levels exhibit a wide range. There have been a number of studies that have demonstrated that even minute variations in respiratory rate can result in hyperventilation. Take into consideration that these alterations may result in symptoms. It has been demonstrated that mathematical computations do not cause an increase in respiration due to stress [16]. Accordingly, the stressors of the experiment need to be analyzed. Stressors, on the other hand, such as horror films, cause an increase in respiration rates because the subject is concerned about the content of the visual stimulus [27]. This concern is expressed by the subject in response to the content of the film. This subsection investigates the stressors that are utilized in the detection of mental stress as well as the effects that these stressors have on physiological parameters. Within the scope of this study, stressors and metabolic fluctuations will be investigated.

As evidence of the Wearable Stress and Affect Detection (WESAD) database’s widespread use, approximately 550 books and other publications have cited it [29]. This particular database contains the Trier Social Stress Test (TSST), which is known to induce stress, and it provides a dataset for research purposes. Stress is caused by the TSST, which comprises elements such as speech and arithmetic modules. Both modules are included in the experimental protocol. To evaluate the effects that interruptions and time pressure have on physiological parameters, these conditions can be incorporated into study designs [30,31].

Depending on the objectives of the applications, the stressors that are utilized are determined, and the participant’s reaction to stress is evaluated at each phase. For example, Khomidov et al. investigated whether or not the levels of stress affect the amount of cognitive load. Following the completion of the procedure, an experimental model was developed. In this model, participants in the study are subjected to a variety of activities that are designed to induce cognitive stress. Stress in the workplace was investigated in the majority of these tasks. The employees were distracted from important tasks, received an excessive amount of emails, and gave presentations without prior planning [32]. This research project ought to be able to identify significant variations in heart rate and heart rate variability (HRV) through the utilization of facial analysis and camera-based remote photoplethysmography (rPPG). The investigation revealed that there was a significant difference from the values that were considered the baseline. Whenever tasks required a fight-or-flight response, they discovered that HRV decreased while HR increased. French et al. [33] investigated the utilization of emotional film clips as a means by which patients suffering from eating disorders could recognize stress. A total of four videos, each lasting three minutes, were shown to the participants to elicit feelings of fear, happiness, and melancholy. Immediate stress-related elements were discovered through the fear films test, relative to other activities. Monitoring oscillations in skin conductance and respiration yielded the desired results [33].

### 3.3. Questionnaires

Validated questionnaires for mental stress assessment allow researchers to link subjective mental states with physiological data. This integration supports the development of future mental health monitoring systems with improved accuracy. Commonly used questionnaires are examined and compared for their contribution to advances in mental stress identification.

Spielberger et al. [14] created the State-Trait Anxiety Inventory (STAI), which is used in universities and hospitals to assess anxiety. For example, one study evaluated whether social media addiction causes anxiety in nursing students. In addition, STAI has been combined with machine learning to predict stress based on physical activity and smoking behavior [1,17]. STAI works with physiological parameters like heart rate and pupil diameter to classify chronic stress levels [16]. The STAI can assess state and trait anxiety, making it useful for behavioral and physiological marker research, as it captures both transient and stable components of anxiety.

The Perceived Stress Scale (PSS-10), developed by Cohen et al., is reliable for measuring stress [15], as demonstrated in extensive research. Research in Brunei and Bosnia supports this evidence of reliability and validity. These studies show that the scale can distinguish positive and negative stress responses, supporting its use in real-time, general-purpose stress monitoring systems [34,35].

Other questionnaires include Siegrist et al.’s Effort–Reward Imbalance (ERI) Questionnaire [36]. This questionnaire examines the imbalance between effort and reward. Several studies, including one in Sweden, have confirmed the ERI. These studies linked a high effort–reward imbalance to mental stress and lower job satisfaction. However, its applicability is limited to workplace situations, which restricts its usefulness in non-work settings [37].

The Job Content Questionnaire (JCQ) by Karasek et al. [38] and the Copenhagen Psychosocial Questionnaire (COPSOQ III) by Llorens et al. [39] are workplace-specific occupational stress measures. The JCQ has been validated, for example, in Vietnam among hospital nurses. The COPSOQ III, validated in a large German population, assesses workplace psychosocial stress effectively but has limited generalizability [40,41].

Lovibond et al. developed the Depression Anxiety Stress Scales (DASS-21) to quickly assess stress, anxiety, and depression [42]. The validity and reliability of the original DASS-42 are maintained in this 21-item version. This version’s practical and time-efficient structure makes it suitable for therapeutic and research use. The DASS-21 was designed to evaluate overlapping psychiatric symptoms through three subscales targeting distinct emotional domains. Recently published research by Al-Dassean et al. (2024) confirmed the Jordanian DASS-21’s three-factor structure [43]. In Arabic-speaking populations, the DASS-21 has good psychometric properties. This study showed that the test is adaptable to different cultures, making it a global indicator of psychological distress. Accordingly, the DASS-21 is widely used for measuring mental health in various settings due to its simple format, cross-cultural validation, and strong psychometric properties.

These surveys combined with physiological data in real-time computerized systems have improved mental health monitoring. STAI-based machine learning models and chronic stress detection systems have shown that integrating subjective data and physiological markers can improve stress prediction and validate stress levels in real-world scenarios [16,17].

### 3.4. AI Algorithms

The key approach to assessing the validity of research results is to examine how they are interpreted. Modern technology uses trained artificial intelligence (AI) algorithms to classify and compute this information automatically. Robust pipelines ensure that the appropriate machine learning model is selected and evaluated throughout feature extraction and classification. AI algorithms have changed the way data is processed in several academic fields. However, effective use requires computational competence to avoid missing important information through an inappropriate classification approach or strategy. This section reviews selected AI systems for evaluating mental stress factors and their capability to categorize stress states in real time.

The K-Nearest Neighbor algorithm (KNN), a simple supervised classifier, predicts the class of data points based on proximity. Accordingly, the data points do not follow a predetermined distribution based on the algorithm’s training phase. KNN adapts to the observed data and classifies samples using distance-based metrics. Consequently, when new data is processed, the algorithm can assign it to the appropriate class. Numerous factors affect the expression of mental stress, which can be problematic because higher dimensionality increases the likelihood of classification errors. This can also occur when features are highly redundant or noisy, which alters the proximity range of the selected “k” value and leads to poor performance [2]. Therefore, the “k” value must be carefully selected because the likelihood of underfitting can increase with its size. Ultimately, KNN performance depends on dataset quality, with more discriminative data leading to distinct and more accurate classification. Hemakom et al. used KNN in a study comparing mental stress variability across genders using ECG and EEG signals [8]. The authors initially used only ECG features to categorize the data (non-stress vs. low/high stress). Next, they employed KNN to attain the following accuracy: 73.25% for mixed genders, 78.48% for females, and 73.77% for males. However, when both ECG and EEG physiological signals were considered, performance dramatically improved to 87.5%, 90.95%, and 88.87%, respectively, indicating improved class separability. The authors also discuss how the algorithm can effectively interpret data in real time, demonstrating the utility of KNN for data processing in experimental analysis [8,30].

Random Forest (RF) is another algorithm that is well-known for its capacity to perform accurate data classification. It uses decision trees with randomly chosen features to determine the classification using a majority-vote method. Unlike KNN, RF does not rely on a “best-fit line”; instead, it aggregates decisions across multiple trees to classify data according to behavioral patterns connected to each class. RF is often computationally demanding to design and modify because of its ensemble nature. Nevertheless, after training, RF can be one of the most effective classification algorithms used in research. Its binary classification processing time supports its applicability in real-time data processing [10]. Al-Alawi et al. successfully classified stress levels with an accuracy of 99.5%, while Gideon Vos et al. produced encouraging results with an accuracy of 93% [2]. These studies report consistently strong accuracy.

To efficiently classify data points, the Support Vector Machine (SVM) uses an optimal separating hyperplane. After training, the algorithm separates classes based on feature similarities. This mechanism allows the algorithm to select a suitable kernel function (linear, nonlinear, exponential, radial basis function (RBF), etc.) to separate and categorize the data points appropriately. Notably, results may depend on the kernel function chosen, particularly when classification performance varies with data dimensionality. In this regard, two studies conducted by Zainudin et al. and Kang et al. [7,11] found that an SVM using the RBF kernel produced contrasting accuracies (79% vs. 88.9%) when validating and classifying mental stress levels. Although they both employed RBF, the datasets may differ in separability and noise, which can increase the risk of misclassification and may be mitigated by selecting a different kernel function. In general, using SVM alone is not typically associated with the best performance in real-time applications [6].

Alternatively, a stacking classifier is an ensemble approach that combines the strengths of several methods to optimize results throughout data collection and inference. A classification algorithm is frequently combined with an optimization algorithm to maintain high accuracy and processing speed. Gupta et al. conducted an experimental investigation comparing the performance of SVM plus the Modified Whale Optimization Algorithm (MWOA) to SVM alone. The results reveal a significant increase in classification performance, with accuracy rising by 13.76% by combining MWOA with the original SVM method [9].

Unsupervised learning is becoming more popular as AI technology advances, resulting in algorithms that can train themselves to understand and process data while also adapting to evolving standards and needs. However, neural networks are not inherently unsupervised; they can be trained in supervised, unsupervised, or self-supervised settings. Conceptually, neural networks are inspired by the human brain, with interconnected units (neurons) collaborating to evaluate and discover patterns that explain the data. Furthermore, artificial neural networks (ANNs) have several channels built into their design to facilitate decision-making by allowing the algorithm to access information from throughout the network. Unlike the aforementioned techniques, ANN may improve performance as datasets grow in size by identifying patterns and connecting the data to representations inside the network. However, computational strain can be substantial and costly. Nonetheless, by selecting a suitable architecture, classification can be performed efficiently with promising results. Gondowijoyo et al. reported that ANNs can categorize factors related to mental stress detection with accuracy up to 95% [12].

Deep learning, a subset of machine learning, extends neural-network modeling through architectures such as convolutional neural networks (CNNs) and recurrent neural networks (RNNs) to enable efficient data processing and classification. CNNs primarily focus on visual data, such as image recognition, processed across numerous layers. CNN layers, unlike ANNs, are not entirely linked; they are typically organized into convolutional (feature extraction) and pooling (dimensionality reduction) layers, which help reduce feature confusion in high-dimensional data. Barki et al. investigated a 4-block CNN architecture composed of convolutional, pooling, and dropout layers, which contributed to a classification accuracy of 92.04% [44]. Furthermore, RNNs are used for sequence-based data, where iterative connections are included in the network design. RNNs can manage new inputs by referencing and cross-validating previously computed information, which is especially useful for time-series data. Long Short-Term Memory (LSTM) networks are a form of RNN that uses memory cells to address limitations of standard RNNs, such as vanishing gradients. Thus, LSTMs can be applied to long-term sequence modeling. Moser et al. evaluated LSTM for identifying mental stress, reporting an average accuracy of 98.3% [45].

### 3.5. Sensors

Wearable devices for detecting mental stress and recording physiological signals linked to stress reactions are designed using a variety of sensors, as shown in Figure 3. These include GSR, EEG, ECG, HRV, HR, PD, PWA (Pulse Wave Amplitude), and SpO_2_. Each sensor provides distinct data dimensions that, when combined, result in a comprehensive stress profile.

GSR sensors assess variations in skin conductance in response to emotional arousal, making them particularly useful for stress detection. For instance, the Grove 1.2 GSR sensor demonstrated high sensitivity and accuracy during the COVID-19 pandemic. It successfully distinguished between university students’ calm and stressed states during simulated exams [30,46]. Additionally, GSR systems connected to LabVIEW provided continuous real-time monitoring, making them suitable for wearable technology. Integrating GSR with HR and PD produced a high sensitivity of 91.7% and accuracy of 89.7% in one chronic stress study, demonstrating GSR’s role in real-time stress monitoring [16].

ECG devices can identify stress by analyzing autonomic responses and cardiac activity, enabling the detection of stressed individuals. In the context of wearable devices, the AD8232 sensor was capable of producing high-quality ECG with three leads [47]. In a subsequent study, ECG and electromyogram (EMG) signals were collected using electrodes made from biocompatible potato peels [48]. This experiment demonstrated that such electrodes enable real-time signal quality monitoring, as reflected in PWA observations. Heart rate variability (HRV) represents autonomic homeostasis and is a widely used stress biomarker. HRV is associated with stress, making it an excellent candidate for wearable stress monitoring [49,50]. This correlation was confirmed through meta-analysis. Additional research [51] highlighted the importance of standardizing HRV measurements for stress monitoring to improve measurement precision. The HRV functionality of wearable devices enables continuous, non-invasive stress assessment, improving overall system efficiency and user acceptance [52].

Heart rate and oxygen saturation are also indicators of stress. A wearable system that tracked heart rate, breathing rate, and motion cadence enabled multi-modal stress detection [53]. Al Abdi et al. corroborated the effectiveness of heart rate (HR) in chronic stress classification when combined with galvanic skin response (GSR) and pupil diameter (PD), establishing HR as a crucial element in real-time monitoring [16]. When combined with SpO_2_, HR can reflect stress-related changes in circulatory and respiratory function. PWA is a cardiovascular marker that can capture stress responses by distinguishing between stressful and non-stressed conditions. When used with GSR, HR, and PD, PWA improves real-time system reliability for multi-sensor stress tracking [16]. AI may further improve HRV-based stress monitoring [54]. Cheng et al. observed that deep learning techniques, such as CNNs, enhance the accuracy of remote photoplethysmography (rPPG) heart rate monitoring.

Pupil diameter (PD) is increasingly used to quantify mental stress and cognitive workload. Task-evoked pupil responses were associated with cognitive effort in healthcare training, underscoring the significance of PD for real-time monitoring [55]. Another study demonstrated that pupil measures enhance HR and GSR in stress detection systems by capturing cognitive exertion through PD and HRV [52]. Al Abdi et al. [16] reported that PD is highly effective for detecting and quantifying stress, indicating its suitability for wearable stress-monitoring systems. PD is often measured using infrared cameras, which enable real-time tracking of pupil–iris contrast. Pupil dilation is associated with cognitive workload and stress, making this modality effective for evaluating cognitive load [56].

EEG sensors are effective for assessing mental states. An EEG study of olfactory and cognitive responses confirmed the EEG’s capability for real-time stress detection. Power within specific EEG frequency bands, together with HRV, indicated mental ease and engagement [57]. This study supports integrating EEG and ECG data into wearable technologies to enhance stress detection, particularly for real-time monitoring.

Secondary indicators such as body temperature and respiratory rate (RR) are utilized to detect stress. For optimal monitoring, RR should be used alongside HRV, HR, GSR, and EEG [58]. One study [59] reported no significant association between body temperature and stress, suggesting that it may be less informative for stress monitoring. Tutunji et al. reported that combining physiological data from wearable biosensors with mood measurements in machine learning models improved stress categorization, supporting multi-sensor stress detection for personalization [60].

## 4. Comparison of Reviewed Techniques and Methods

In this section, a comparison and analysis of the methods and procedures utilized in stress detection are presented. Techniques of stress detection, stressors, questionnaires, artificial intelligence algorithms, and sensing modalities are all compared and contrasted in this section. The purpose of this comparison is to highlight the strengths and weaknesses of each method, as well as each method’s capacity to evaluate mental stress in real time. This comparison identifies effective combinations and trends that improve accuracy, reliability, and practical deployment in applications that are used in the real world. Using this comparison, we hope to identify effective combinations and practical trends.

### 4.1. Comparative Evaluation of Detection Techniques

Context is extremely important when comparing and evaluating techniques that are invasive and those that are non-invasive. It is necessary to take into consideration the setting, the number of subjects, the duration, and the objectives of the experiment when selecting a methodology. This helps to justify the methodological choice. Table 1 contains a number of articles that share a common objective: the system should be able to detect stress and ensure that the user is comfortable. This goal is typically given higher priority. In terms of interpretation, complexity, applicability, advantages, and disadvantages, the various methods of data extraction are completely different from one another. Nevertheless, in the field of research, it is not always sufficient to eliminate particular approaches based on a single variable that is absent. For experimental analysis to be successful, it is necessary to improve feature selection while simultaneously reducing the complexity of computation.

In terms of real-world applicability, participant compliance is a decisive constraint: methods that require clinical procedures or produce discomfort are less suitable for repeated or continuous monitoring, even if their biochemical measurements are highly precise. Conversely, wearable approaches trade some signal purity for usability; therefore, improving sensor–skin interfaces and reducing motion-related noise is critical to maintaining data quality without sacrificing comfort. Recent work on ultrathin, skin-like dry electrodes demonstrates how electrode material and adhesion design can reduce noise and improve signal stability during dynamic monitoring, supporting more reliable long-term acquisition of electrophysiological signals in practical settings [26].

When conducting experiments, it is strongly recommended and widely practiced to steer clear of invasive methods because of the negative effects they have. In order to lessen the influence that external factors have on the data collection process, experimental settings ought to be controlled and technologically advanced. This characteristic is likely to be present in applications involving venipuncture. It is more likely that errors and cross-contamination will occur when manual methods are used. Consequently, the efficiency of the experiment is diminished, which places the participants in needless danger [23,61]. As a result of the development of more sophisticated monitoring technologies, the use of such methods in experimental trials is increasingly difficult to justify.

Concerns regarding user security and data collection can, however, be addressed through the use of noninvasive procedures. When it comes to improving feature quality, wearable sensor applications are more flexible than invasive procedures that measure cortisol levels. This is even though conventional procedures produce more consistent data. Code development allows for a wide variety of different ways in which software modifications can be made. The training and boosting algorithms are included in these modifications. When we choose the appropriate sensors, we are able to highlight the physiological signals that are pertinent to our research, which in turn has an impact on the process of feature extraction. A further significant component in the execution of the process is the overall processing pipeline. A GSR, PPG, EEG, and ECG are the most important sensors for detecting stress and non-stress parameters [6,18,62]. Experiments of this kind may take into account the safety and comfort of the participants, generate accurate results if the computations are completed correctly, and, most importantly, analyze data effectively, given that noninvasive procedures do not involve any obstructions.

### 4.2. Comparative Analysis of Stressors

When physiological parameters and stressors are taken into consideration, it is possible to compare different methods of stress induction. Mathematical computations might not effect on the rate of respiration. On the other hand, emotionally charged stimuli such as horror films almost always cause an increase in respiration because of the distressing effects they have. Aligning the stressor with observed characteristics is important to match the stressor to the targeted physiological response to accurately measure physiological changes. This comparison reveals distinct patterns, indicating that some stressors may have more general restrictions that reduce their effectiveness. While certain stressors are particularly effective at focusing on certain physiological responses (such as HR and HRV during public speaking), this comparison also suggests that different stressors preferentially activate different physiological pathways, mainly because particular stressors effectively target particular physiological responses.

Identifying mental stress can be accomplished through the use of stressors such as memory and cognitive issues, as well as the fight-or-flight response. A list of the stressors can be found in Table 2. It is through the activation of the sympathetic nervous system or the hormone cortisol that these strategies cause stress. With the help of this strategy, the desired outcomes can be accomplished. Before beginning the process, it is important to select an appropriate stressor in order to guarantee that there will be sufficient physiological signal fluctuations used for feature extraction and stress level classification. The Trier Social Stress Test (TSST) will be found in the WESAD database [29]. This test is well-known for its ability to induce stress through activities such as public speaking and time-pressured mathematics. These kinds of tests, which are known to cause stress, are commonplace. This method is appropriate for research on stress because it incorporates external stressors such as interruptions, which have the potential to cause significant changes in both the HR and the HRV. For the purpose of stress research, the method is appropriate. The use of pre-existing datasets, despite the fact that it is convenient, severely restricts the investigation of novel stressors or hybrid techniques [63].

In the field of experimental research, gaining access to relevant datasets continues to be a significant obstacle. A significant number of researchers make use of WESAD [29], which is both a dataset and a documented method for stress induction. Both of these aspects are essential to the research process. On the other hand, stressors that are designed for particular research objectives and sensors might produce more specific insights overall. Khomidov et al. demonstrate that stressors can be tailored to the requirements of particular sensors. They found significant HR and HRV fluctuations with cognitive and public-speaking stressors by using remote PGG (rPPG) face analysis because of the correlation between the two. The factors that caused these changes were stressors. To provide further clarification, HR increased while HRV decreased, which is indicative of a fight-or-flight response [32].

It was demonstrated by French et al. that emotionally charged films can cause stress. The researchers discovered that films that induced fear had a greater impact on changes in respiratory arrhythmia and skin conductance than films that were either happy or sad. Furthermore, these findings highlight the significance of selecting stressors that correspond to the physiological characteristics that are being investigated [33]. This ensures that stress levels are differentiated and classified in the appropriate manner.

While each stressor offers unique insights, inappropriate selection or inadequate stress levels can hinder experiments, reducing the effectiveness of feature extraction and subsequent analyses. Because of this, the focus of future research ought to be on hybrid stressors, which incorporate aspects of a number of different methodologies currently in use. This will assist in comprehending the physiological changes that are brought about by stress.

### 4.3. Comparison of Questionnaires

A comparison of the strengths and weaknesses of the mental stress detection questionnaires that were investigated is presented in Table 3. Depending on their intended use, these questionnaires can be classified as either general, clinical, or occupational. The following provides a concise summary of the primary characteristics of the questionnaire, including its applicability, strengths, and weaknesses. In addition to this, it offers suggestions for how these components can be incorporated into wearable monitoring systems for mental health.

The use of generic questionnaires such as the STAI and PSS-10, which are able to detect both acute and chronic stress and have been validated for application across a variety of populations, is one trend that has emerged. These examinations have been validated for a wide variety of patients. In the context of wearable healthcare systems, the STAI has the capability to measure both state and trait anxiety, which makes it an effective tool for detecting stress in real time [1,16,17]. It is for the same reason that the PSS-10 is able to detect stress in educational and medical settings [34,35]. For this particular purpose, it is suitable due to its high reliability and consistent results across a wide range of demographics.

Occupational stress questionnaires, such as the JCQ, ERI, and COPSOQ III, prioritize work-specific stressors and offer useful data regarding job-related stress. However, their design restricts their use outside of professional settings. For instance, the JCQ and ERI assess factors like effort–reward imbalance and job demands, but their work-centric design limits their broader applicability [36,39,64]. Similarly, COPSOQ III has shown effectiveness in assessing psychosocial stress at work, but it cannot be modified for general stress monitoring outside of the workplace [41]. Therefore, these tools are more appropriate for occupational health studies than real-time, cross-context stress monitoring in wearable systems.

The strengths and weaknesses of the mental stress questionnaires that were found to be effective are presented in Table 3. Whichever purpose the questionnaires are intended for—general, clinical, or occupational—will determine the strengths and weaknesses of the questionnaires. Included in this is a discussion of the potential incorporation of each questionnaire into wearable mental health monitoring systems, as well as a summary of the most important aspects of each questionnaire, such as its applicability, strengths, and weaknesses.

The STAI, PSS-10, and DASS-21 are some of the assessments that are especially helpful for generalization. Their adaptability, ease of scoring, and cross-cultural validation make them ideal for use in a range of contexts. Their broad demographic applicability increases their potential for wearable stress monitoring systems [1,16,17,34,35]. The DASS-21’s compact structure, which provides a comprehensive mental health assessment, can be practical for wearable systems intended for general mental health monitoring [43].

Examples of specialized occupational questionnaires that provide thorough insights into work-related stress are the ERI and JCQ. However, their limited applicability outside of work settings limits their usefulness in wearable systems. The ERI offers unique insights into long-term work-related stress [36], but the JCQ and COPSOQ III are great instruments for evaluating job control and psychosocial work conditions [40,41]. Although these benefits are helpful for workplace research, wearable technology integration is challenging with longer, more complex formats like COPSOQ III.

Though not designed with work-related stressors in mind, the STAI and PSS-10 are extremely useful in a broad mental health assessment. On the other hand, although the DASS-21 measures a number of mental health issues, it does not have the depth to assess work-related stressors as done in the JCQ and ERI.

Overall, the STAI and PSS-10 are the two most flexible tools to date for a generalized assessment of mental stresses and can be easily used for the incorporation of physiological measurement capabilities in wearable devices. On the contrary, even if the JCQ, ERI, and COPSOQ III are a precious source of information for occupational health issues, their scope and design constraints render them less flexible for generalized purposes and time-sensitive stresses. These findings indicate that a generalized form of questionnaires such as the STAI and PSS-10, taken together with the incorporation of physiology, can lead to a holistic and flexible technique for real-time stress measurement in wearable devices.

### 4.4. Performance Comparison of AI Algorithms

As previously stated, the AI algorithm used for data processing has a direct impact on the capacity to provide useful and comprehensible outcomes. To guarantee familiarity with the conceptual elements relevant to each model and to permit comparison between their performances in real-time applications, the information supplied and adequate research are therefore crucial. Features including structural and computational complexity, real-time application, accuracy, and overall performance, as shown in Table 4, enable us to rule out particular algorithms based on how well they identify stress. It is noteworthy that additional algorithms, including decision trees (DTs), linear regression (LR), and Naive Bayes (NB), were also taken into consideration. They were not within our inclusion criteria due to poor performance when independently employed in mental stress detection [11,68].

The chosen parameters to observe mental stress levels are physiological signals offering the most prominent changes between emotional states. Hence, by eliminating the risk of inaccuracies imposed by incorrect sensor readings or artifacts disrupting the data quality, AI algorithms can perform classification by observing the presented data. However, mental stress expression differs notably between individuals, resulting in outliers that the algorithm may not accurately identify. For instance, algorithms like KNN or RF, which rely on proximity to classify the data, are more susceptible to misclassifying outliers when deducing the most probable output [8,13]. On the other hand, an ANN can self-train and assess the classification by referencing background information relevant to stress. This mechanism promotes consistently accurate results by generating the outcome through learned decision boundaries rather than probability [68].

**Figure 4:**
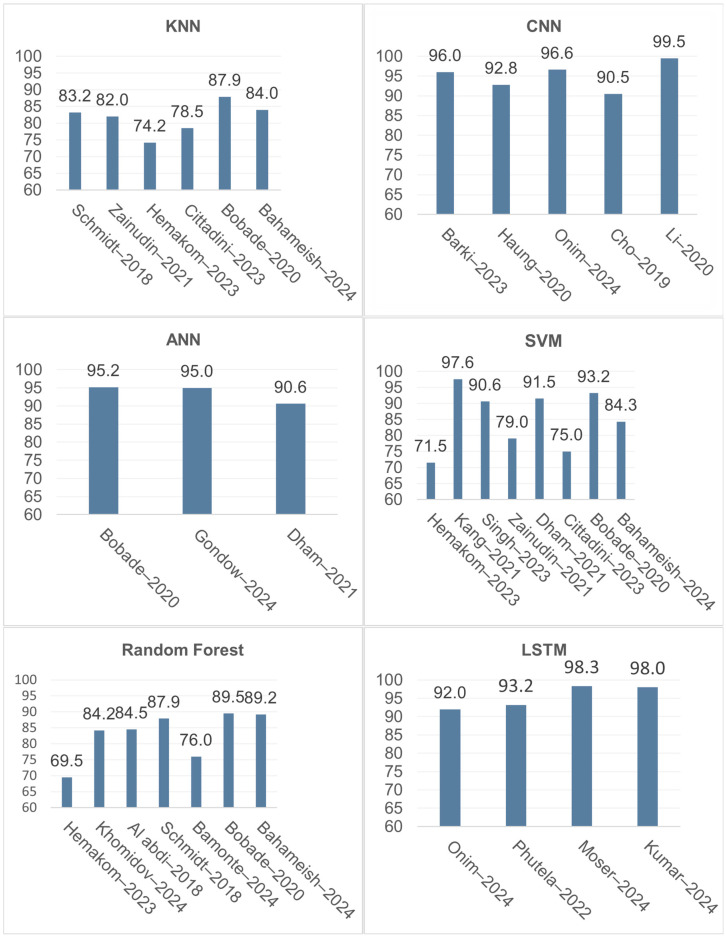
Depiction of accuracy variation between different AI algorithm applications in the literature: KNN, Random Forest, SVM, ANN, CNN, LSTM [7,8,11,12,16,20,29,32,44,45,64,65,66,70,72,74,75,76,77,78,79].

Conversely, the benefits proposed by the preceding algorithms can be utilized in constructing an optimal model, also known as an ensemble model. Ensemble models have been employed in several research articles discussed in Table 5, where any variation in algorithms can be used to enhance data processing. SVM in conjunction with ANN and SVM in conjunction with WOA were the most successful stacking classifiers [9,29]. These two models produced accurate outlier categorization, consistent separation between observed parameters, and proficient accuracy. These characteristics confirm why ensemble models are exceptional in every facet of data processing techniques, adequately fulfilling all requirements. Conversely, a significant drawback is the degree of complexity involved in configuring and computing ensemble models, which can be addressed by improving our comprehension of such applications. A comparison of AI algorithm applications in the literature is presented in Figure 4, where accuracy performance is emphasized appropriately.

### 4.5. Comparative Assessment of Sensors

Several physiological sensors are utilized in published research to identify instances of mental stress, as demonstrated in Table 6. Each sensor has its own set of advantages and disadvantages, and it can be utilized in a varied range of contexts. Due to the close relationship that they have with the autonomic nervous system, HRV, ECG, and GSR are considered to be primary metrics. Because of their close connection to the system, they are optimal for monitoring stress levels. The real-time detection of stress is improved by these sensors, which are frequently found in wearable devices. Through the provision of multidimensional stress insights, they enhance the detection of stress.

The respiratory rate (RR) is an effective stress indicator because breathing patterns have a strong correlation with the levels of stress that an individual faces. Using noninvasive respiratory analysis for wearables, Jarchi et al. [82] demonstrated that reflectance was an essential component. Wearables that use PPG are able to estimate the respiratory rate in a variety of body locations. Because of recent developments in PPG, its precision and dependability have been improved in a variety of contexts. A PPG-based RR estimation technique developed by Iqbal et al. was demonstrated to be accurate for use with wearable devices [61]. For noninvasive stress measurement, PPG-based RR monitoring is not only practical and accurate, but also user-friendly.

Integration of physiological data improves the accuracy of stress detection, despite the fact that RR is significant. A multimodal PPG-based stress monitoring method was proposed by Paul et al. [81], which uses HRV in conjunction with other physiological measurements in order to monitor stress. A real-time HRV and PPG signal interpretation system that is image-based and uses machine learning was developed [62,81]. This methodology was continuously refined by the researchers. Wearable technology has recently demonstrated the advantages of combining inputs to improve stress detection.

Through the detection of sympathetic nervous system activity, GSR can identify stress. Inaccurate results may be obtained, however, due to factors such as movement and environmental conditions such as temperature and humidity. While it helps detect stress, it does have some limitations. ECG sensors, particularly three-lead ones, are highly effective stress indicators when combined with heart rate variability (HRV) analysis. These sensors monitor the activity of the heart. When it comes to three-lead sensors, this is specifically true. On the other hand, precise positioning is necessary, which can be challenging in real-world situations.

Several studies have demonstrated that simple metrics can serve as a supplement to more complex markers such as HR and oxygen saturation (SpO_2_). We recommend using these metrics with markers that are more complicated. While these non-invasive and low-cost measures may not be as specific as HRV, they do contribute to real-time monitoring in wearable products. By measuring the diameter of the pupil and the activity of the brain, electroencephalography (EEG) and pupillometry can reveal the amount of mental effort and stress that is being exerted. But their applicability in the real world is limited by the fact that they are sensitive to the environment and the EEG setting is complicated.

In multimodal systems, machine learning makes it possible to create prediction models for stress assessment in real time. Within the context of continuous stress monitoring, Namvari et al. [62] conducted an evaluation of wearable PPG devices to demonstrate their usefulness. Machine learning makes it possible to conduct predictive real-time assessments. There is a stress-prediction dataset that was developed by Iqbal et al. [61]. RR and other physiological measures were utilized in this dataset.

However, despite the positive developments, real-time processing of multiple signals is a challenging task. To be worn for extended periods of time, wearable technology needs to be both comfortable and unobtrusive. Efforts to reduce the size of the device and developments in sensor performance under a variety of conditions will be of critical importance. It is possible to improve the accuracy of stress-detection systems by combining physiological and behavioral data, which will also make devices more user-friendly.

Stress detection can be improved with wearable sensors like PPG, GSR, ECG, HRV, HR, and SpO_2_, especially in multimodal information systems that use these sensors and machine learning. These studies demonstrate the potential for wearable stress monitoring devices to improve accuracy, comfort, and adaptability in various situations.

## 5. Discussion

The growing body of research reviewed in this paper highlights the increasing importance of stress detection and monitoring within occupational and everyday environments. Advances in embedded sensing technologies, wearable devices, and artificial intelligence have enabled the development of increasingly sophisticated systems capable of capturing stress in real time [2,13,83,84]. This section synthesizes the key findings from the reviewed studies, discusses their implications, and outlines existing challenges and opportunities for future research.

**Figure 5:**
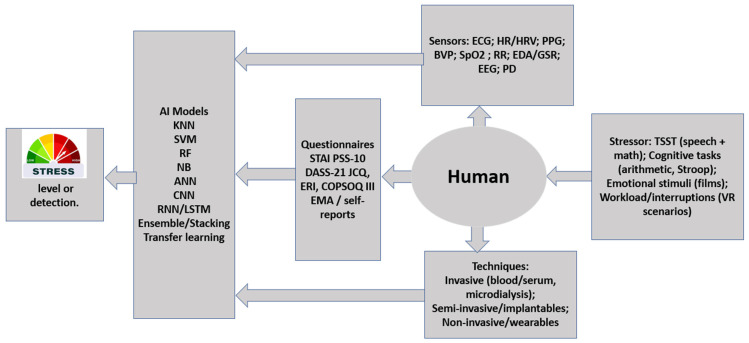
Overview schematic of wearable mental stress detection and monitoring.

Figure 5 summarizes the wearable mental-stress detection workflow reviewed in this paper, linking stressors to physiological responses captured by multimodal sensors, supported by questionnaires/self-reports for labeling and validation. It also highlights how AI models transform these inputs into stress detection or stress-level estimation outcomes.

Throughout the reviewed literature, several practical limitations were identified. First, signal quality and data management can be challenging in wearable stress monitoring, where recordings may be affected by noise, missing segments, and variability in measurement conditions. Second, algorithm performance is often inconsistent across studies because different datasets, protocols, and labeling strategies are used, which complicates direct comparison and model selection. This limitation is particularly evident when models are evaluated on small or homogeneous datasets, leading to limited generalizability in real-world settings [2,16,60,80,83]. In studies that include EEG, additional challenges arise due to the complexity of setup and the need for extensive preprocessing, as EEG is more susceptible to contamination from eye blinks and environmental interference [8,9,65]. Overall, these factors contribute to performance variations and highlight the need for robust preprocessing, standardized protocols, and cross-dataset validation [31,68,83].

Despite these challenges, there is potential for future research. First, there is a need for noise-resistant sensors that are designed for wearable technology and are more accessible. Modifying the design of a wearable device’s sensor can enhance its functionality and precision. Enhancing sensor designs can lead to a reduction in EEG interference [60,74]. Another possibility is to explore additional stress indicators [75,85]. Additionally, employing more advanced preprocessing techniques and flexible algorithms capable of managing diverse datasets may enhance the robustness and generalizability of stress detection AI models [64,83]. These directions provide a strong foundation for further progress. Utilizing standardized datasets or implementing transfer learning may assist in reducing disparities, thereby enhancing AI-driven stress detection [29,71]. These advancements could notably improve wearable device efficacy, enabling more accurate and adaptable stress-monitoring solutions in real-world environments.

The increasing focus on multimodal stress detection techniques, which integrate multiple physiological markers including GSR, heart rate variability (HRV), and pupil diameter, is one of the main advantages of the current research [70]. It has been demonstrated that combining several biomarkers can improve classification accuracy [76]. For instance, compared to using single-modality inputs, machine learning models that combine GSR and HRV have shown increased precision in recognizing stress levels [29,51]. Although HRV is often considered an excellent candidate for stress detection, its reliability in wearable, real-world settings should be interpreted cautiously. HRV features depend on accurate inter-beat intervals, which can be degraded by motion artifacts, posture changes, and poor sensor–skin contact, potentially biasing HRV estimates and increasing false classifications in free-living conditions [51,60]. Therefore, HRV-based models should incorporate artifact handling and/or multimodal fusion to improve robustness [62,77,83]. Furthermore, stress detection technology may now be integrated into small, wearable devices thanks to developments in miniaturized and low-power biosensors, enabling long-term monitoring with little discomfort for the user [53,54]. AI methods, such as neural networks, have further optimized the analysis of these multimodal signals, enhancing their predictive power [72,86].

At the same time that these benefits are present, there are a great deal of disadvantages. A significant disadvantage is that many of the methods for stress detection do not have sufficient practical validation. Real-world situations are characterized by increased variability due to the presence of motion artifacts, ambient noise, and physiological differences.

A further practical limitation for wearable stress detection is the presence of motion artifacts during real-world use. Movement, changes in posture, and loose sensor–skin contact can introduce substantial noise into wearable signals—particularly PPG/BVP and EDA—leading to distorted inter-beat intervals, unreliable HR/HRV features, and spurious changes in skin conductance. This issue is less prominent in controlled laboratory protocols but becomes critical in free-living environments, where participants naturally walk, gesture, and perform daily tasks, and where ambient conditions also fluctuate [53,60]. Therefore, future wearable stress-monitoring systems should include signal-quality assessment and artifact-aware preprocessing (e.g., filtering, rejection of corrupted segments, and activity-aware correction), and should leverage multimodal sensing (e.g., combining accelerometry and physiological channels) to improve robustness under motion [62,82].

There is a greater degree of variability in real-world situations compared to laboratory-controlled research, which typically produces positive results [55,60]. There is a significant amount of research that makes use of small and consistent sample sizes, which restricts its applicability to various groups. As a research limitation, this is the case. There are psychological and physiological responses to stress that are influenced by factors such as age, gender, health, and lifestyle. Because of the wide range of responses to stress, this is an extremely important aspect of stress research [34,79]. A further challenge that must be considered—particularly in relation to stressor design and interpretation—is inter-individual variability in stress reactivity. Even when the same stressor is applied (e.g., TSST, cognitive tasks, emotional stimuli), physiological responses can differ substantially across individuals due to demographic and physiological factors such as age, sex/gender, BMI, and fitness level. These factors influence baseline autonomic function, endocrine reactivity, and recovery dynamics, which in turn affect the magnitude and temporal patterns of markers commonly used for stress detection (e.g., GSR, HR/HRV, PPG-derived indices, and pupil diameter). Consequently, models trained on homogeneous or small samples may not generalize well to broader populations, and apparent performance differences across studies may partly reflect variations in participant characteristics rather than algorithmic superiority [8,31]. Future work should therefore report participant demographics more consistently and incorporate stratified evaluation (e.g., by age group, gender, BMI category, and fitness level) and/or adaptation strategies to improve robustness and reduce bias in real-world deployment [2,83]. The stress detection models that are based on artificial intelligence need to take these variations into account. It is necessary to modify algorithms to make them applicable to a wider range of circumstances [66,80].

The computing requirement of advanced stress detection methods is an additional notable issue. For instance, deep learning models may not be feasible for real-time wearable applications because of their high training data requirements and processing demands [64]. The requirement for lightweight, efficient algorithms that can operate on edge devices with constrained processing capacity is highlighted by the ongoing trade-off between model complexity and real-time processing performance [5,25].

These results have implications that go beyond advancements in technology. Wearable technology that incorporates accurate stress detection has the potential to completely transform methods for monitoring and intervening in mental health issues. Digital health platforms, for example, might incorporate real-time stress monitoring to give consumers individualized stress management advice and instant feedback [40,51]. This could be particularly advantageous in high-stress situations, like the workplace or school, when prompt responses could lessen long-term physiological and psychological effects [21,69,87].

Additionally, enhancing the accessibility and dependability of stress detection systems may make it easier to conduct extensive epidemiological research on illnesses linked to stress. Researchers may be able to better understand stress patterns and develop more focused mental health treatments and public health policies by gathering and evaluating ongoing physiological data from a variety of populations [54,60]. AI could play a central role in automating and scaling such analyses, making it easier to identify trends and at-risk populations [28,80].

Future research should prioritize the creation of standardized stress datasets that include a wide range of environmental and demographic factors in order to fill in the gaps that have been identified in the existing body of knowledge. In addition, accelerating the field would require the establishment of standards for stress detection algorithms, thereby enabling more informative cross-study comparisons [34]. Professionals in the fields of engineering, psychology, and medicine must collaborate in order to accomplish the development of stress detection systems that are both scientifically accurate and applicable [83]. Research on wearable stress detection has made progress, but there are still obstacles that prevent its widespread application. It is necessary to overcome challenges such as sensor noise, inconsistent datasets, and computing constraints. There is a possibility that in the future, wearable stress monitoring will improve the management of mental health and well-being. Enhanced sensor technology, trustworthy artificial intelligence models, and validation in the real world could be used to address these limitations.

## 6. Conclusions

**Figure 6:**
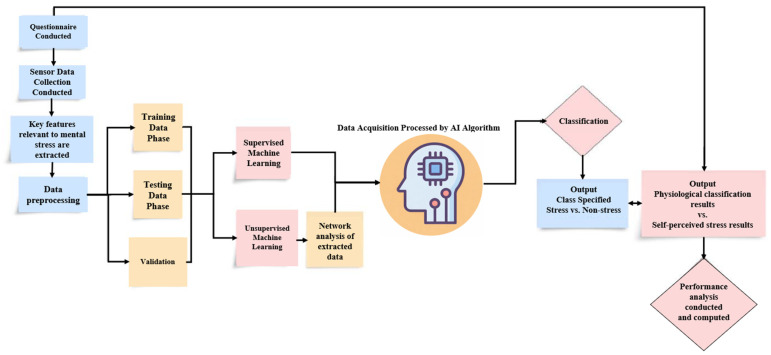
System workflow block diagram illustrating the process of mental stress detection using AI and machine learning. Data are collected through questionnaires and physiological sensors, followed by feature extraction and preprocessing.

A better understanding of the primary goals and mechanisms behind the mental stress detection strategies shown in Figure 6 was made possible by the framework that was provided by our literature study. The majority of mental illnesses that affect workers and students are brought on by stress. Neglecting stress can lead to feelings of anxiety and depression. It is possible to initiate early intervention through the detection of mental stress by conducting research into how situational stresses influence physiological markers that are associated with well-being. As a result, early intervention is possible. There has been a significant amount of information regarding mental stress that has been gathered through the use of non-invasive wearable sensors such as EEG, ECG, and GSR. With the help of these sensors, mental stress and its effects on health were quantified. To increase real-world impact, future work should prioritize a small set of practical research gaps. First, the most promising wearable setups for scalable deployment are multimodal. They should combine one strong autonomic marker with another complementary channel. A practical example is EDA/GSR plus PPG or ECG (HR/HRV features). Adding an accelerometer is also recommended, as it can help. Pupillometry can further improve detection of cognitive stress. However, it works best when lighting is controlled or can be normalized. Second, reliable AI classification requires minimum data-quality standards, including explicit signal-quality indices (e.g., contact quality and SNR), artifact flags (motion/posture changes), and standardized reporting of sampling rate, window length, and missing-data handling, otherwise reported accuracies may not translate to free-living use. Third, transfer learning should be applied concretely via pretraining on large public datasets and fine-tuning on small target cohorts, and performance should be validated with cross-dataset testing rather than single-dataset splits. Finally, workplace deployment raises ethical requirements: transparent consent, purpose limitation (health support—not surveillance), secure storage, and clear governance to prevent punitive use. Studies should also evaluate fairness across demographic groups and report mitigation steps. Addressing these priorities would improve generalizability and readiness for continuous monitoring beyond laboratory protocols.

## Figures and Tables

**Table 1:** Summary of invasive and non-invasive stress detection techniques and their performance, limitations, and real-time applicability.

Source	Technique Type	Technique Method	Performance	Drawbacks	Availability and Complexity	Real-Time Applicability	Consistency
[22] 2023	Invasive	Salivary Biosensor/Dental Augmentation	Biosensor shows accurate cortisol measurement in saliva.	Requires surgical implantation; uncomfortable; interference with dental structures.	Requires professional expertise; costly to implement.	Time-consuming initially; real-time once implanted.	Produces consistent cortisol level results correlating with stress.
[21] 2023	Invasive	Blood Sampling	Provides sufficient cortisol-level data from blood.	Uncomfortable; cross-contamination risk.	Requires anatomical expertise and proper needle handling.	Takes time for lab results; hours needed.	Cortisol levels are measured accurately in response to stress variations.
[23] 2024	Invasive	Serum Sampling and Microdialysis	Serum sampling is accurate; microdialysis tracks cortisol precisely.	Uncomfortable due to venipuncture; microdialysis needs specialized equipment.	Serum extraction is time-consuming; microdialysis probes need careful placement.	Real-time is possible after microdialysis probe insertion.	Both methods offer consistent cortisol and stress correlation.
[25] 2024	Non-Invasive	Physiological Sensors (ECG, GSR, EDA, PPG, BCG)	85% accuracy; 86% specificity.	Inadequate sensors can cause inconsistent data collection, affecting accuracy.	Easily accessible; easy to use; data widely available for proper sensor utilization.	Applicable and does not require much computational power or time.	Different sensors illustrate different physiological signals; the importance of proper sensor selection.
[27] 2024	Non-Invasive	Physiological Sensors (EEG, SCR, ST, HRV)	Sufficient variability to classify stress vs. non-stress scenarios.	Inadequate sensors may cause inconsistent data collection.	Easily accessible; easy to use; some sensors cost-ineffective (e.g., EEG).	Applicable and does not require much computational power or time.	Appropriate results showing stress variation; some sensors outperform others.
[28] 2021	Non-Invasive	Thermal Imaging	With increased stress, a consistent increase in RAVs in pixels. Visual data validated by pulse and blood pressure changes.	Temperature variability can be impacted by the surrounding environment.	Easily accessible; affordable; requires expertise in data preprocessing.	Applicable and does not require much computational power or time.	Variable consistency where stress spikes vary by individual; RAVs increased consistently with stress.

**Table 2:** Comparative analysis of commonly used stress-induction methods in the literature.

Source	Stressor	Parameters	Advantages	Disadvantages
[5,29]	TSST	BVP, ECG, EDA, EMG, RESP, TEMP	Extremely reliable for inducing stress. Enhanced stress-inducing tasks like public speech. Correlates self-perceived stress with physiological signals simultaneously.	Artificial environment may impact generalizability. Ethical concerns regarding participant discomfort. Limited contextual relevance.
[8,11]	Arithmetic solving	ECG, EEG	Cognitive load correlates well with stress. Standardized methodology. Easy to compute and extract features.	Not applicable for extreme stress fluctuations. Limited variability.
[9,64]	Image recognition	EEG	Immediate feedback for stress level variations. High accuracy and reliability. Easily integrated for feature extraction.	Cognitive overload may cause detection errors. Data skew is possible due to individual differences. Requires a precise training phase.
[44,65]	Stroop color-word test	HR, HRV	Immediate physiological variations are observable. Widely used and standardized.	Performance is influenced by language literacy. Non-native speakers may yield inconsistent results.
[33,66]	Stress-inducing films	EEG, ECG, GSR, PPG	Films involving horror/anxiety trigger stress fluctuations. Easy to access; unaffected by external factors due to content nature.	Subjective, which may lead to ungeneralizable data. Time-consuming; some films last up to 1 h.

**Table 3:** Analysis of common questionnaires for mental stress assessment.

Article Reference	Questionnaire	Key Findings	Applicability to Mental Stress Detection	Strengths	Limitations
[1,14]	State-Trait Anxiety Inventory (STAI)	Used to assess anxiety levels in various settings, including academic stress and chronic stress detection.	High applicability for detecting both acute and chronic stress through anxiety assessment.	Measures both state and trait anxiety, reliable across contexts.	Limited to anxiety, not as comprehensive for other stress types.
[34,35]	Perceived Stress Scale (PSS-10)	Demonstrated high validity and reliability in diverse groups, differentiates positive and negative stress indicators.	Broad applicability in mental stress detection across different demographics.	Generalizable, reliable across demographic and cultural variations.	May not capture specific workplace stressors like JCQ or COPSOQ.
[38,40]	Job Content Questionnaire (JCQ)	Effective in evaluating work-related stress in hospital settings, adaptable within job-focused environments.	Limited applicability to general mental stress detection; focused on occupational stress.	Well-suited for workplace stress, captures job-specific stressors.	Not suitable for non-occupational stress; lacks generalizability.
[39,41]	Copenhagen Psychosocial Questionnaire (COPSOQ III)	Valid and reliable for assessing psychosocial stress in large-scale occupational samples.	Primarily applicable to occupational stress; less relevant for general mental stress.	Effective for large-scale workplace studies, reliable performance.	Inflexible for non-work environments, lengthy for wearable use.
[36,67]	Effort-Reward Imbalance (ERI)	Measures perceived imbalance between work effort and rewards; linked to chronic stress and job dissatisfaction.	High applicability to workplace stress but limited in non-occupational settings.	Focuses on chronic stress due to work imbalances, valid in occupational studies.	Primarily occupational, not generalizable outside workplace stress contexts.
[42,43]	Depression Anxiety Stress Scale (DASS-21)	Validated in multiple languages; measures depression, anxiety, and stress in a unified format.	Moderate to high applicability; useful for comprehensive mental health assessment.	Combines multiple mental health indicators, concise and effective.	General stress scale; not specialized for specific settings.

**Table 4:** Comparative analysis of selected AI models for mental-stress detection, summarizing model complexity, real-time applicability, key limitations, and main strengths reported in the reviewed literature.

Source	Model	Complexity	Real-Time Applicability	Limitations	Strengths
[8,10]	KNN	Simple and easy to use. Does not require much computation if dataset is small.	Viable	Not effective for large datasets. Sensitive to outliers. Prone to inaccuracies due to proximity-based classification.	Simple in design and use. Only initial training data is required. Flexible in applications.
[10,64]	RF	Relies on decision trees. More trees = higher complexity, especially with multidimensional data.	Viable	Complex ensemble design. Time-consuming to train and set up. Heavy computational load.	Highlights important/relevant features. Can handle missing data. Maintains accuracy.
[9,10,11,69]	SVM	Simple once data is extracted. Key factor: ensuring the right kernel function.	Viable	Computationally taxing. Prone to noise and overlapping class confusion. Relies heavily on kernel function selection.	Less prone to overfitting. Kernel functions flexible for non-linear data.
[12,65,70]	ANN	Inherently complex. Requires expertise. After selecting correct architecture, ANN can self-train.	Viable	Requires large datasets. Complex architecture. High risk of overfitting. Computationally taxing.	No training phase needed after architecture selection. Effective classification. Learns from patterns.
[44,71]	CNN	Multilayer architecture. Complex design. Requires modern computational tools.	Viable	Computationally taxing. Prone to overfitting. Requires large datasets.	Automatic feature extraction. High accuracy. Effective for complex data.
[45,72]	LSTM	Multilayer architecture. Time-consuming to configure.	Viable	Complex architecture. Better performance with large datasets. Limited long-sequence handling. Prone to overfitting.	Mitigates vanishing gradient in RNNs. Effective for sequence-based classification. Pattern referencing.
[64,65,69,73]	Ensemble Models	Variable complexity depending on chosen algorithms.	Viable	Complex design and computation. Time-consuming. May overload data depending on base models.	More accurate than single models. Less prone to noise. Less overfitting. Flexible in applications.

KNN: K-Nearest Neighbors, RF: Random Forest, SVM: Support Vector Machine, ANN: Artificial Neural Network, CNN: Convolutional Neural Network, LSTM: Long Short-Term Memory (A Type of RNN), RNN: Recurrent Neural Network.

**Table 5:** Comparative performance of AI models for mental stress detection across representative studies (binary classification accuracy).

Ref/Year	Dataset Size	Physiological Modality (Detailed)	Stressors/Experimental Tasks	Classification Model	Primary Accuracy
[3] 2019	19 subjects	EDA, ST	Lab + real-world stress	Rule-based	84%
[11] 2021	31 ECG records	ECG (HRV intervals)	Picture, Video, Stroop, Math	SVM + NB	97.6%
[7] 2021	252 subjects	ECG, EDA	Stress tasks	DT, SVM, KNN, MLP	95%
[12] 2024	20 subjects	ECG + EEG	Arithmetic + visual stimulus	ANN	95%
[9] 2020	14 subjects	EEG	Cognitive stress tasks	Modified SVM	96.4%
[16] 2018	60 subjects	PD, ECG, EDA, Respiration, Skin Temperature	Chronic stress assessment (validated using Perceived Stress Scale)	RF, NB	89.7%
[44] 2023	14 subjects	In-ear PPG	Stroop + arithmetic + cold pressor	1D CNN	96.0%
[45] 2024	28 subjects	EDA + Skin Temp	Audio stimulus	LSTM	98.3%
[29] 2018	15 subjects	ECG, EMG, EDA, RR, Temp, BVP	TSST	DT, RF, AB, LDA, KNN	93.1%
[74] 2021	15 subjects	ECG, EMG, Temp, BVP	TSST	Ensemble	99.9%
[72] 2024	39 subjects	EDA, BVP, Temp, RR	TSST	RF, CNN	92.5%
[66] 2022	35 subjects	EEG	Emotional video clips	LSTM	93.2%
[75] 2024	150 subjects	Speech	Psychological stress speech	LSTM	98%
[76] 2024	30 subjects	PPG + GSR	Emotional film clips	RF, SVM	76%
[76] 2020	15 subjects	Multimodal (WESAD)	TSST	ANN	95.2%
[70] 2023	2001 samples	ST + EDA	Physical/mental stress	RF, GB	99.5%
[64] 2020	15 subjects	Multimodal (WESAD)	TSST	1D CNN	99.5%
[77] 2024	38 subjects	ECG-derived HRV	Cognitive stress task	RF, KNN, SVM	89%

ECG: electrocardiogram; EEG: electroencephalography; EDA: electrodermal activity; GSR: galvanic skin response; EMG: electromyography; PPG: photoplethysmography; BVP: blood volume pulse; HRV: heart rate variability; RR: respiration rate; ST: skin temperature; AB: AdaBoost, NB: naïve Bayes; DT: decision tree; GB: gradient boosting; LDA: linear discriminant analysis; WESAD: Wearable Stress and Affect Detection dataset; TSST: Trier Social Stress Test; WESAD database link [29].

**Table 6:** Summary of physiological sensing modalities used in mental stress detection, highlighting key findings, deployment applicability, strengths, and limitations across the reviewed studies.

Article Reference	Physiological Parameters	Key Findings	Applicability to Mental Stress Detection	Strengths	Limitations
[16,45,46,70,76,80]	Galvanic Skin Response (GSR)	Effective in distinguishing stress from relaxation.	High applicability detects stress through physiological arousal.	Reliable; real-time monitoring.	Environment-sensitive; calibration needed.
[7,11,12,57,64,70,77]	Electrocardiogram (ECG)	Reliable for real-time heart activity monitoring.	High applicability for autonomic response tracking.	Accurate and supports chronic stress monitoring.	Motion artifacts; placement sensitivity.
[16,53,60,78]	Heart Rate (HR) and Oxygen Saturation (SpO_2_)	Reflects stress-related cardiovascular changes.	Moderate applicability for general stress monitoring.	Non-invasive; low cost.	Limited in stress-specific insights.
[16,52,55]	Pupillometry (PD)	Detects mental workload and cognitive stress.	High applicability; indicates cognitive load.	Non-invasive; complements HRV/GSR.	Affected by lighting conditions.
[9,12,57,66]	Electroencephalogram (EEG)	Correlates brain activity with stress levels.	High applicability for cognitive assessment.	Direct brain activity insights; strong for cognitive tasks.	Complex and intrusive setup.
[16]	Pulse Wave Amplitude (PWA)	Monitors cardiovascular stress responses.	High applicability for chronic stress detection.	Useful in multi-sensor systems.	Sensitive to posture.
[16,59,64,70]	Respiratory Rate (RR)	Monitors breathing rate changes related to stress.	Moderate applicability; supplementary stress measure.	Easy to integrate.	Less reliable alone; affected by physical activity.
[16,44,76,81]	Photoplethysmography (PPG)	PPG-based systems achieve high accuracy in stress monitoring.	High applicability; non-invasive monitoring of stress indicators.	Provides HR, HRV, RR; supports wearable integration.	Noise and motion sensitivity.

## Data Availability

The original contributions presented in this study are included in the article. Further inquiries can be directed to the corresponding author(s).
